# A consensus building exercise to determine research priorities for silver trauma

**DOI:** 10.1186/s12873-020-00357-4

**Published:** 2020-08-21

**Authors:** Abdullah Alshibani, Jay Banerjee, Fiona Lecky, Timothy J. Coats, Rebecca Prest, Áine Mitchell, Emily Laithwaite, Matt Wensley, Simon Conroy

**Affiliations:** 1 Department of Health Sciences, College of Life Sciences, University of Leicester, Leicester, LE1 7HA UK; 2 Emergency Medical Services Department, College of Applied Medical Sciences, King Saud bin Abdulaziz University for Health Sciences, Riyadh, Saudi Arabia; 3 University Hospitals of Leicester NHS Trust, Leicester, UK; 4 Centre for Urgent and Emergency Care Research, University of Sheffield, Sheffield, UK; 5 Emergency Medicine Academic Group, Department of Cardiovascular Sciences, University of Leicester, Leicester, UK; 6 Sligo University Hospital, Sligo, Ireland

**Keywords:** Injury, Geriatrics, Older adults, Emergency, Prehospital, Research questions, Trauma, Triage

## Abstract

**Background:**

Emergency care research into ‘Silver Trauma’, which is simply defined as major trauma consequent upon relatively minor injury mechanisms, is facing many challenges including that at present, there is no clear prioritisation of the issues. This study aimed to determine the top research priorities to guide future research.

**Methods:**

This consensus-based prioritization exercise used a three-stage modified Delphi technique. The study consisted of an idea generating (divergent) first round, a ranking evaluation in the second round, and a (convergent) consensus meeting in the third round.

**Results:**

A total of 20 research questions advanced to the final round of this study. After discussing the importance and clinical significance of each research question, five research questions were prioritised by the experts; the top three research priorities were:

1. What are older people’s preferred goals of trauma care?
2. Beyond the Emergency Department (ED), what is the appropriate combined geriatric and trauma care?
3. Do older adults benefit from access to trauma centres? If so, do older trauma patients have equitable access to trauma centre compared to younger adults?

**Conclusion:**

The results of this study will assist clinicians, researchers, and organisations that are interested in silver trauma in guiding their future efforts and funding toward addressing the identified research priorities.

## Background

‘Silver Trauma’ is major trauma consequent upon relatively minor injury mechanisms, commonly seen in older people. As the population of older adults is increasing worldwide [1, 2], so will rates of silver trauma. Emergency and prehospital care represent central parts of a trauma patients’ care journey, and play major roles in determining their outcomes [3–5]. The growth of the silver trauma population has resulted in increased demand to provide the optimal level of emergency care which, in turn, requires more evidence-based practice for the treatment and management of this population. However, research into silver trauma is not extensive and many research questions remain unanswered [6, 7]. Furthermore, it is unclear which outcomes might be valued most by older people with silver trauma – whose priorities might well be different to working age population (the main beneficiaries of trauma research to date) [8, 9].

An effective way to determine research priorities in healthcare is to build consensus amongst clinicians. A well-recognised approach is the Delphi technique, which has been used in a wide range of clinical areas [10–17]. However, no such study has been conducted looking into silver trauma. Therefore, the aim of this study was to build a consensus among clinicians to determine research priorities for silver trauma.

## Methods

This research priority consensus exercise applied a three-step modified Delphi technique. The process of this study consisted of an idea generating (divergent) first round, a ranking evaluation in the second round, and a (convergent) consensus meeting in the third round. The full protocol of this study is already published [8]. The quality indicators and, in addition, the methodological criteria for reporting Delphi studies in publication were considered when developing the study protocol [18].

### Study protocol

In the first round, a web-based questionnaire using Google Forms was generated and sent via email. The participants from the designated networks in this study were asked to compose up to three research questions they considered important to address in future research. Research questions should be written according to Patient, Intervention, Comparison, and Outcome (PICO) format and submitted within a two-week period. In addition, demographic data was collected from the participants in this round and in all the rounds in this study. Ideas that described the same issue and research question were grouped. Issues that were not related to silver trauma and others that lacked the main components of a PICO format to develop a research question were excluded by the study team.

The second round of this study consisted of two steps. In the first step, a ‘Silver Trauma Panel’ consisting of geriatricians and emergency physicians from the United Kingdom (UK), with a self-identified interest in silver trauma was formed to evaluate the questions from round I. All geriatricians and emergency physicians from Leicester Royal Infirmary (LRI) hospital, National Health Service (NHS), UK who are involved in the treatment and management of older trauma patients were invited to be members of the panel. This step was added to the predetermined study protocol in order to reduce the number of the research questions to a manageable number before launching the next step of this round. Clear and specific meeting schedule and inclusion criteria were predetermined before the meeting. After that, a face to face meeting was held to discuss each research question and decide if it was clinically significant and needed to be addressed in future research. Questions which at least 50% or more of the silver trauma panel voted as ‘Yes’ to be clinically significant to improve the outcomes of this population and needing to be addressed in future research were included in the next step of round II.

In the second step of this round, a second web-based questionnaire using Google Forms was sent through email to all members of the designated networks in this study which remained online for two weeks. The invited participants were asked to prioritise each research question from the first step of round II on a 5-point Likert scale according to their perceived level of importance [19]. Research questions reaching inclusion or non-consensus thresholds in this step were predetermined to progress to the final round of the study. The predetermined consensus thresholds of both steps are shown in Table 1.

**Table 1:** Round II predetermined consensus thresholds

**Round II (a) Consensus thresholds**	
**Inclusion**	≥ 50% of the votes with ‘Yes’
**Exclusion**	> 50% of the votes with ‘No’
**Round II (b) Consensus thresholds**	
**Inclusion**	> 75% of respondents provide a positive result (four or five) on the Likert scale for all criteria.
**Exclusion**	> 75% of respondents provide a negative result (one or two) on the Likert scale for all criteria.
**Non-consensus**	When the proposed priority research question has met neither the inclusion nor exclusion consensus thresholds.

In the third round of this study, a consensus meeting was held where experts in trauma care for older people from East Midlands Major Trauma Network (EMMTN) – Frailty Group, UK were invited to discuss the results of round II of the study. After that, each expert was asked to rank the top three research questions that they thought most important using the predetermined round III ranking scale (first priority [3 points], second priority [2 points], and third priority [1 point]). The responses from the experts were then collected and analysed for final ranking of the research priories. Research priorities were ranked from the highest to lowest median. In case two or more research priorities had the same median, they were ranked from shorter to wider inter-quartile range. After that, the top three research questions with the highest median and narrowest inter-quartile range were determined and formed the final results of this study.

### The invited participants in all rounds of the study

The online questionnaires in round I and second step of round II of this study were sent through email to all members of the following networks and groups: (1) Trauma Audit and Research Network (TARN) - Older People’s group, UK, (2) The Royal College of Emergency Medicine (RCEM) - Clinical Studies group, UK, (3) The National Ambulance Service Medical Directors (NASMeD), UK, (4) European Geriatric Medicine Society (EuGMS), Europe, (5) American College of Emergency Physicians (ACEP) - Geriatric Emergency Medicine Section, United States of America (USA), (6) Society for Academic Emergency Medicine (SAEM) - Academy of Geriatric Emergency Medicine (AGEM), USA, (7) American Academy of Emergency Medicine (AAEM) - Geriatric Interest Group, USA, and (8) Australasian College for Emergency Medicine (ACEM), Australia. The ‘Silver Trauma Panel’ in the first step of round II of the study composed of geriatricians and emergency physicians from LRI hospital, NHS, UK. In the final round of this study, experts from the EMMTN – Frailty Group, UK, who have strong interest and experience in caring for silver trauma patients were invited to the consensus meeting.

### Data analysis

The percentage of the demographics of the participants in all rounds of the study was calculated. Furthermore, the percentage of the responses to each research question was calculated in step (a) and (b) of round II in this study according to the consensus thresholds of each step. In the final round of this study, the median and interquartile range of each research question, ranked by the experts, were calculated and the top three research priorities, with highest mean and narrowest interquartile range, were determined and constituted the final results of the study.

### Ethical approval

Ethical approval was obtained from the University of Leicester. The invited participants in round I and round II received a participant information sheet which includes the main information of the study and the required tasks to complete the online questionnaires. After reading this, they were asked to agree for their anonymised information to be shared when reporting the survey results if they wish to go ahead and complete the questionnaires. In round III of the study, written consent was obtained from all participating experts at the consensus meeting. Anonymised data was secured in a protected secure network to which only the study team members had access.

## Results

### Demographics

In round I, a total of 442 clinicians were invited through email, of which 94 (21%) participated and completed the online survey. The majority of the participants in this round were doctors *n* = 52 (55%) and paramedics *n* = 39 (42%). Most of the participants were specialised in prehospital care 44 (47%), emergency medicine 41 (44%), and both 4 (4%). Participants had a wide experience and most of them were from the UK *n* = 59 (62%) and the USA 28 (30%) (Table 2).

**Table 2:** Summary of Demographics of the Participants in the Study

Variable	Results ***n*** (%)			
	Round I	Round II (a)	Round II (b)	Round III
**Clinical Role**				
Doctors	52 (55%)	6 (100%)	83 (95%)	4 (50%)
Paramedics	39 (42%)	0	2 (2%)	0
Researchers	3 (3%)	0	1 (1%)	0
Epidemiologist	0	0	1 (1%)	0
Advanced Clinical Practitioners (ACP)	0	0	0	3 (38%)
Nurse	0	0	0	1 (13%)
**Specialty**				
Prehospital Care	44 (47%)	0	4 (5%)	0
Emergency Medicine	41 (44%)	2 (33%)	78 (90%)	6 (75%)
Prehospital Care & Emergency Medicine	4 (4%)	0	0	0
Geriatric Medicine	3 (3%)	4 (67%)	1 (1%)	0
Anaesthesia	1 (1%)		1 (1%)	0
Prehospital Care, Emergency Medicine, and Geriatric Medicine	1 (1%)	0	0	0
Trauma and orthopaedics	0	0	3 (3%)	0
Emergency Medicine, and Geriatric Medicine	0	0	0	1 (13%)
Nursing Management	0	0	0	1 (13%)
**Years of Experience**				
1–5 Years	8 (9%)	0	3 (3%)	0
6–10 Years	14 (15%)	1 (17%)	11 (13%)	1 (13%)
11–15 Years	13 (14%)	0	15 (17%)	3 (38%)
16–20 Years	20 (21%)	3 (50%)	20 (23%)	0
21–25 Years	16 (17%)	0	18 (21%)	2 (25%)
26–30 Years	9 (10%)	1 (17%)	7 (8%)	1 (13%)
over 30 Years	14 (15%)	1 (17%)	13 (15%)	1 (13%)
**Country of Current Clinical Practice**				
United Kingdom (UK)	59 (62%)	6 (100%)	40 (46%)	8 (100%)
United States of America (USA)	28 (30%)	0	23 (26%)	0
Germany	1 (1%)	0	0	0
Finland	1 (1%)	0	0	0
Belgium	1 (1%)	0	0	0
Portugal	1 (1%)	0	0	0
Czech Republic	1 (1%)	0	1 (1%)	0
Switzerland	0	0	1 (1%)	0
Ireland	0	0	1 (1%)	0
Australia	1 (1%)	0	16 (18%)	0
Trinidad and Tobago.	1 (1%)	0	1 (1%)	0
New Zealand	0	0	3 (3%)	0
Canada	0	0	1 (1%)	0

In Round II (a), a ‘Silver Trauma Panel’ consisting of six doctors (geriatricians and emergency physicians) from the UK, with a self-identified interest in silver trauma was formed to evaluate the questions from round I (Table 2).

In round II (b), a total of 562 participants were invited through email. Of the 562 participants, 87 (16%) responded and completed the online survey. Most of the participants in this round were doctors 83 (95%) and most specialised in emergency medicine *n* = 78 (90%). As with round I, participants had a wide range of experience and the majority were from the UK *n* = 40 (46%) and the USA *n* = 23 (26%) (Table 2).

In the final round of this study, eight experts from EMMTN - Frailty Group, UK attended the meeting: four doctors, three Advanced Clinical Practitioners (ACP), and one nurse. Most were specialised in emergency medicine (6/8, 75%). The participating experts had different years of experience and all were from the UK (Table 2).

### Interventions

**Fig. 1:**
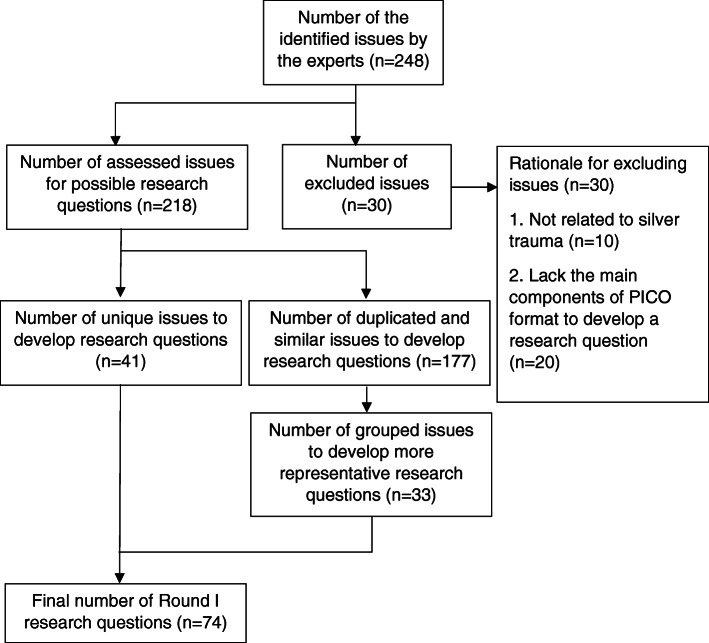
Flow chart of the process of round I analysis

In round I, participants were asked to compose up to three research questions they considered important to address in future research. Ninety-four participants provided 248 issues around improving outcomes of injured older adults. Ideas that described the same issue and research question were grouped. Issues that were not related to silver trauma and others that lacked the main components of the PICO format to develop a research question were excluded by the study team, leaving 74 PICO questions. A flow chart of the process of round I is presented in Fig. 1.

In round II (a), A ‘Silver Trauma Panel’ was convened. The panel had a two-hour face to face meeting to discuss and evaluate the research questions of round I. Of the 74 research questions brought forward from Round I, 20 priorities had at least 50% or more of the votes with ‘Yes’ to be clinically significant and needed to be addressed in future research. Therefore, these 20 research questions were determined to progress to the second step of this round (Table 3).

**Table 3:** Round II (a) determined research priorities

**Research Questions**	
1. What are the possible aspects of cognitive bias that could play a significant role when informing early decisions about timing and mode of imaging and operative interventions for seriously injured older adults? Cognitive bias could include confirmation and availability biases.	
2. Could the development of specific triage criteria improve the outcomes of older trauma patients?	
3. Do older adults benefit from access to trauma centres? If so, do older trauma patients have equal access to trauma centre compared to younger adults?	
4. Beyond the ED, what is the appropriate combined geriatric and trauma care?	
5. Is older abuse effectively assessed among injured older adults in emergency departments?	
6. What is the role of end of life care for older trauma patients in the Emergency and Pre-hospital settings?	
7. What is the appropriate expertise level of provider while caring of injured older adults (ED physician, Trauma surgeon, Academic vs community hospital, designated trauma center vs. not)?	
8. What is the best way for screening and managing rib fractures among older trauma patients to improve their outcomes?	
9. What is the appropriate way to evaluate older adults’ driving competency and skills?	
10. Could the application of “Trauma-geriatric” model similar to “ortho-geriatric” model improve the outcomes of injured older adults?	
11. What is the impact comorbidities including age specific comorbid conditions (like frailty, cognitive decline, and reduced independence) on the outcomes of injured older adults? This could include pain management and secondary trauma.	
12. What is the impact of polypharmacy on the outcomes of injured older adults?	
13. Does the benefits or potential harms of ED geriatric trauma services (diagnostic, therapeutic resuscitation) require quantification as does the value of geriatric-specific ED or geriatric trauma services?	
14. What group of older trauma patients could benefit from trauma team activation?	
15. Is the holistic approach considered by healthcare providers while assessing frail older patients?	
16. How to achieve appropriate, navigable and safe disposition for older trauma patients?	
17. Does patient’s outcomes differ between early Comprehensive Geriatric Assessment (CGA) versus normal care specifically in the setting of a trauma unit or major trauma centre?	
18. What is the most appropriate and effective way to identify spinal injury following low mechanism falls among older people?	
19. What are the older people’s preferred goals of trauma care?	
20. What are the physical and cognitive functional outcomes of older people discharged form hospital following major trauma?	

In round II (b), the participants ranked the 20 research priorities on a 5-point Likert scale. This resulted in seven research priorities meeting the predetermined inclusion threshold (> 75% of the responses were positive [4 or 5 on the Likert scale]), none meeting the exclusion threshold (> 75% were negative [1 or 2 on the Likert scale]), and 13 meeting the non-consensus threshold (met neither the inclusion or exclusion thresholds). Research priorities reaching the inclusion or non-consensus thresholds in this round were predetermined to progress to the final round of this study. Therefore, all 20 research priorities in this round were determined to progress to the final round (Table 4).

**Table 4:** Round II (b) progressing research priorities to the final round of the study

Research Questions	Inclusion (%)	Exclusion (%)
**Included Research Questions**		
1. Could the development of specific triage criteria improve the outcomes of older trauma patients?	86%	7%
2. What group of older trauma patients could benefit from trauma team activation?	82%	6%
3. Could the application of “Trauma-geriatric” model similar to “ortho-geriatric” model improve the outcomes of injured older adults?	79%	6%
4. Do older adults benefit from access to trauma centres? If so, do older trauma patients have equal access to trauma centre compared to younger adults?	78%	6%
5. What is the best way for screening and managing rib fractures among older trauma patients to improve their outcomes?	77%	7%
6. What are the physical and cognitive functional outcomes of older people discharged form hospital following major trauma?	75%	2%
7. Beyond the ED, what is the appropriate combined geriatric and trauma care?	75%	7%
**Non-Consensus Research Questions**		
8. What are the older people’s preferred goals of trauma care?	71%	8%
9. What is the impact comorbidities including age specific comorbid conditions (like frailty, cognitive decline, and reduced independence) on the outcomes of injured older adults? This could include pain management and secondary trauma.	70%	13%
10. What is the impact of polypharmacy on the outcomes of injured older adults?	64%	10%
11. Does patient’s outcomes differ between early Comprehensive Geriatric Assessment (CGA) versus normal care specifically in the setting of a trauma unit or major trauma centre?	63%	8%
12. What is the most appropriate and effective way to identify spinal injury following low mechanism falls among older people?	61%	13%
13. Does the benefits or potential harms of ED geriatric trauma services (diagnostic, therapeutic resuscitation) require quantification as does the value of geriatric-specific ED or geriatric trauma services?	59%	10%
14. What is the role of end of life care for older trauma patients in the Emergency and Pre-hospital settings?	59%	16%
15. How to achieve appropriate, navigable and safe disposition for older trauma patients?	45%	17%
16. Is older abuse effectively assessed among injured older adults in emergency departments?	44%	28%
17. Is the holistic approach considered by healthcare providers while assessing frail older patients?	41%	25%
18. What are the possible aspects of cognitive bias that could play a significant role when informing early decisions about timing and mode of imaging and operative interventions for seriously injured older adults? Cognitive bias could include confirmation and availability biases.	38%	32%
19. What is the appropriate expertise level of provider while caring of injured older adults (ED physician, Trauma surgeon, Academic vs community hospital, designated trauma center vs. not)?	35%	31%
20. What is the appropriate way to evaluate older adults’ driving competency and skills?	33%	46%

The experts in the final round were asked to discuss each of the 20 research questions from round II of the study. After that, each expert was asked to prioritise the top three research questions that they thought most important using the predetermined round III ranking scale. As a result, five of the 20 research questions were prioritised (Table 5), of which the top three were:

1. What are the older people’s preferred goals of trauma care?
2. Beyond the Emergency Department (ED), what is the appropriate combined geriatric and trauma care?
3. Do older adults benefit from access to trauma centres? If so, do older trauma patients have equal access to trauma centre compared to younger adults?

**Table 5:** Round III prioritised research questions

Research Questions		Median	Interquartile Range
1.	What are the older people’s preferred goals of trauma care?	2.5	1.5
2.	Beyond the ED, what is the appropriate combined geriatric and trauma care?	2	0.5
3.	Do older adults benefit from access to trauma centres? If so, do older trauma patients have equal access to trauma centre compared to younger adults?	2	1
4.	What is the best way for screening and managing rib fractures among older trauma patients to improve their outcomes?	2	2
5.	Could the development of specific triage criteria improve the outcomes of older trauma patients?	1	0.5

The experts in this round highlighted the need to determine the appropriate outcome measures specifically for silver trauma patients. One example mentioned by one of the experts was that identifying polytrauma in frail older patients does not always change their outcomes, so in young trauma patients, we usually expect them to walk out of hospital with full functional recovery, while in these patients we may rather look at their comfort and palliation.

## Discussion

Our study invited both national and international clinicians and experts who are interested in silver trauma to discuss and determine the top research priorities for emergency care of silver trauma. Twenty research questions (seven met the inclusion threshold and 13 met the non-consensus threshold) were considered to be important and proceeded to the final round of this study. Of these, the top three research priorities were identified in the final round as follows: (1) What are the older people’s preferred goals of trauma care?, (2) Beyond the ED, what is the appropriate combined geriatric and trauma care?, and (3) Do older adults benefit from access to trauma centres? If so, do older trauma patients have equal access to trauma centre compared to younger adults? The experts at the consensus meeting considered that there is a need to determine the appropriate outcome measures specifically for silver trauma patients, so the impact of emergency care interventions and diagnostics can be accurately assessed for this population.

The study in round I and round II aimed to determine top research questions to improve the outcomes of silver trauma patients that could be applied at Western developed healthcare systems including the UK. As a result, healthcare providers from these systems had participated in forming and prioritising the top research questions (Table 2). However, the study, in round III, focused in determining the top three research questions that could be applied specifically in the UK. This is mainly due to one of the reasons of conducting this study which is determining the top research questions especially in the UK [8]. Therefore, experts in trauma care for older people from the UK only were invited to the meeting in the final round to discuss and determine the top research questions.

This study has some limitations; the response rates in round I and round II were relatively low (21 and 16%, respectively). However, the aim of this study is not to get high response but rather to have a good representation of research ideas, which appeared to be the case. The main focus in this study was to determine silver trauma research priorities in Western developed healthcare systems, so the results of this study may not be applicable to other regions. Although our questionnaires and consensus meeting invitations were sent to networks and groups that involved healthcare professionals from different clinical roles, most of the participants and experts in this study were doctors; other members of the healthcare team may have alternative thoughts. Moreover, although we invited members from EuGMS and some other groups and networks which are interdisciplinary, we had minimal representation from geriatricians in our study. We were not able to collect data on reasons for non-response, so it is difficult to know why geriatrician invitees were under-represented.

The findings from this study, will help researchers, healthcare professionals, and policymakers prioritise funding calls relating to trauma and older people. Important areas for future research might include developing Patient Reported Outcomes for silver trauma, developing and evaluating geriatric attuned trauma services or assessing outcomes for older people with silver trauma who are cared for in trauma centres.

## Conclusion

This study has identified the top research priorities that should be addressed in future research to improve emergency care of silver trauma patients. The top research priorities of silver trauma include determining the appropriate combined geriatric and trauma care beyond ED care, identifying the perspectives of older trauma patients about their preferred goals of their trauma care, and determining the benefits of major trauma centre access for silver trauma patients and assessing the equity of such access if it is determined to be beneficial. The research priorities will be provided to Trauma Audit and Research Network (TARN) in the UK to inform their future silver trauma research and disseminated to funders. Other research questions in the top 20 may be of interest in other parts of the world. Overall, the results of this study could significantly help in improving the quality of emergency care research of silver trauma to be highly effective and towards more patient-centred care.

## Data Availability

The datasets used and/or analysed during the current study are available from the corresponding author on reasonable request.

## Abbreviations

ED: Emergency Department
PICO: Patient, Intervention, Comparison, and Outcome
LRI: Leicester Royal Infirmary
NHS: National Health Service
UK: United Kingdom
EMMTN: East Midlands Major Trauma Network
TARN: Trauma Audit and Research Network
RCEM: Royal College of Emergency Medicine
NASMeD: National Ambulance Service Medical Directors
EuGMS: European Geriatric Medicine Society
ACEP: American College of Emergency Physicians
USA: United States of America
SAEM: Society for Academic Emergency Medicine
AGEM: Academy of Geriatric Emergency Medicine
AAEM: American Academy of Emergency Medicine
ACEM: Australasian College for Emergency Medicine
ACP: Advanced Clinical Practitioners
